# The genome sequence of the Eurasian minnow, *Phoxinus phoxinus *(Linnaeus, 1758)

**DOI:** 10.12688/wellcomeopenres.22867.1

**Published:** 2024-09-03

**Authors:** Andy D. Nunn, Paolo Moccetti, Bernd Hänfling, Wellcome Sanger Institute Tree of Life Management, Samples and Laboratory team, Wellcome Sanger Institute Scientific Operations: Sequencing Operations, Wellcome Sanger Institute Tree of Life Core Informatics team, Tree of Life Core Informatics collective, Darwin Tree of Life Consortium

**Affiliations:** 1 Department of Natural Sciences, University of Hull, Hull, England, UK; 2 Institute for Biodiversity and Freshwater Conservation, University of Highlands and Islands, Inverness, Scotland, UK

**Keywords:** Phoxinus phoxinus, Eurasian minnow, genome sequence, chromosomal, Cypriniformes

## Abstract

We present a genome assembly from an individual female *Phoxinus phoxinus* (the Eurasian minnow; Chordata; Actinopteri; Cypriniformes; Leuciscidae). The genome sequence spans 950.50 megabases. Most of the assembly is scaffolded into 25 chromosomal pseudomolecules. The mitochondrial genome has also been assembled and is 18.36 kilobases in length.

## Species taxonomy

Eukaryota; Opisthokonta; Metazoa; Eumetazoa; Bilateria; Deuterostomia; Chordata; Craniata; Vertebrata; Gnathostomata; Teleostomi; Euteleostomi; Actinopterygii; Actinopteri; Neopterygii; Teleostei; Osteoglossocephalai; Clupeocephala; Otomorpha; Ostariophysi; Otophysi; Cypriniphysae; Cypriniformes; Cyprinoidei; Leuciscidae; Phoxininae; *Phoxinus*, *Phoxinus phoxinus* (Linnaeus, 1758) (NCBI:txid58324).

## Background

The Eurasian minnow *Phoxinus phoxinus* (L.) has a wide geographical distribution that includes the majority of Europe and large parts of northern Asia ( Kottelat & Freyhof, 2007). It is small, invariably less than 10 cm in length, and generally resident in fresh water ( Maitland, 2004), although some populations inhabit brackish environments ( Svirgsden *et al.*, 2018). Age-at-maturity, size-at-maturity and life span are typically 1–2 years, 4–5 cm and 3–4 years, respectively, but there is considerable variation across its range ( Maitland, 2004; Mills, 1988). The taxonomy and systematics of the genus are complex and unresolved, and the current species name probably includes more than one distinct taxonomic entity ( Cheng *et al.*, 2022; Denys *et al.*, 2020; Freyhof & Kottelat, 2008).

It is a rheophilic species, so predominantly inhabits rivers, but can also occur in connected still waters ( Frost, 1943; Nunn *et al.*, 2007a). Typical habitats include glides in the middle reaches of rivers, pools in upland streams and the margins of lowland rivers ( Mann, 1996; Prenda *et al.*, 1997; Watkins *et al.*, 1997). Eurasian minnow are potamodromous, with small-scale migrations sometimes occurring prior to the spawning period, but the majority of individuals in most populations are otherwise comparatively sedentary ( Frost, 1943). However, temporal and ontogenetic shifts in habitat use, such as diurnal movements between river margins or tributaries and the main channel to forage, to shallow water to spawn or coinciding with the transition from the larval to the juvenile period, have been documented ( Garner *et al.*, 1998; Nunn *et al.*, 2010; Simonović *et al.*, 1999; Watkins *et al.*, 1997).

Eurasian minnow are sexually dimorphic, and this is exaggerated during the spawning season when males develop breeding tubercles and a distinctive colouration ( Denys *et al.*, 2020; Maitland, 2004). The species is iteroparous, and spawning can occur as single or multiple events over the spring and summer ( Mills, 1987; Nunn *et al.*, 2007c), with females each depositing ~200-3000 eggs on clean gravel, usually in highly oxygenated, flowing water ( Frost, 1943; Mann, 1996; Mills, 1988). Larvae, juveniles and adults consume mainly small crustaceans, insect larvae and algae ( Frost, 1943; Maitland, 2004; Nunn *et al.*, 2007b), and growth rates are positively influenced by water temperature and highest in the first year of life ( Mills, 1988).

According to the International Union for the Conservation of Nature (IUCN) Red List of Threatened Species, Eurasian minnow is classified as “Least Concern” in terms of extinction risk as its abundance and geographical range do not meet the criteria to qualify as threatened ( Freyhof & Brooks, 2011; Freyhof & Kottelat, 2008; Nunn *et al.*, 2023). Notwithstanding, the species faces localised threats due to a range of widespread pressures, especially pollution, habitat degradation and excessive fish stocking ( Freyhof & Kottelat, 2008).

Here we present a chromosomally complete genome sequence for *Phoxinus phoxinus*, based on one female specimen from the River Wharfe, UK. Another chromosomal level genome sequence for this species (GCA_037504875.1 and GCA_037504845.1) was recently released by the Leibniz Institute for the analysis of biodiversity change.

## Genome sequence report

**Figure 1.**
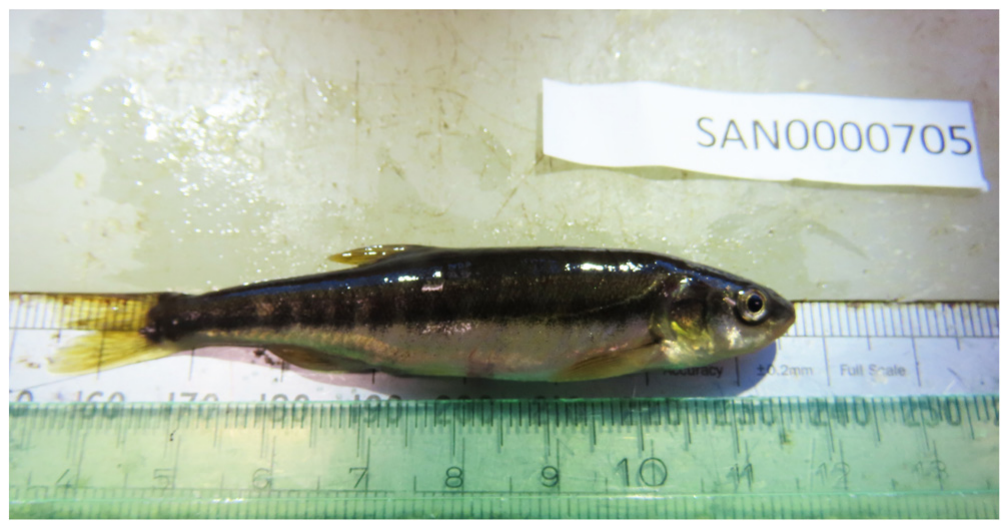
Photograph of the *Phoxinus phoxinus* (fPhoPho1) specimen used for genome sequencing.

The genome of an adult *Phoxinus phoxinus* ( Figure 1) was sequenced using Pacific Biosciences single-molecule HiFi long reads, generating a total of 29.23 Gb (gigabases) from 2.41 million reads, providing approximately 30-fold coverage. Primary assembly contigs were scaffolded with chromosome conformation Hi-C data, which produced 117.37 Gbp from 777.27 million reads, yielding an approximate coverage of 123-fold. Specimen and sequencing information is summarised in Table 1.

**Table 1.** Specimen and sequencing data for *Phoxinus phoxinus*.

Project information			
**Study title**	Phoxinus phoxinus (Eurasian minnow)		
**Umbrella BioProject**	PRJEB59308		
**Species**	*Phoxinus phoxinus*		
**BioSample**	SAMEA11296539		
**NCBI taxonomy ID**	58324		
Specimen information			
**Technology**	**ToLID**	**BioSample accession**	**Organism part**
**PacBio long read sequencing**	fPhoPho1	SAMEA11296601	spleen
**Hi-C sequencing**	fPhoPho1	SAMEA11296600	gill
**RNA sequencing**	fPhoPho1	SAMEA11296606	muscle
Sequencing information			
**Platform**	**Run accession**	**Read count**	**Base count (Gb)**
**Hi-C Illumina NovaSeq 6000**	ERR10818326	7.77e+08	117.37
**PacBio Sequel IIe**	ERR10812863	2.41e+06	29.23
**RNA Illumina NovaSeq 6000**	ERR11242520	7.74e+07	11.69

**Figure 2.**
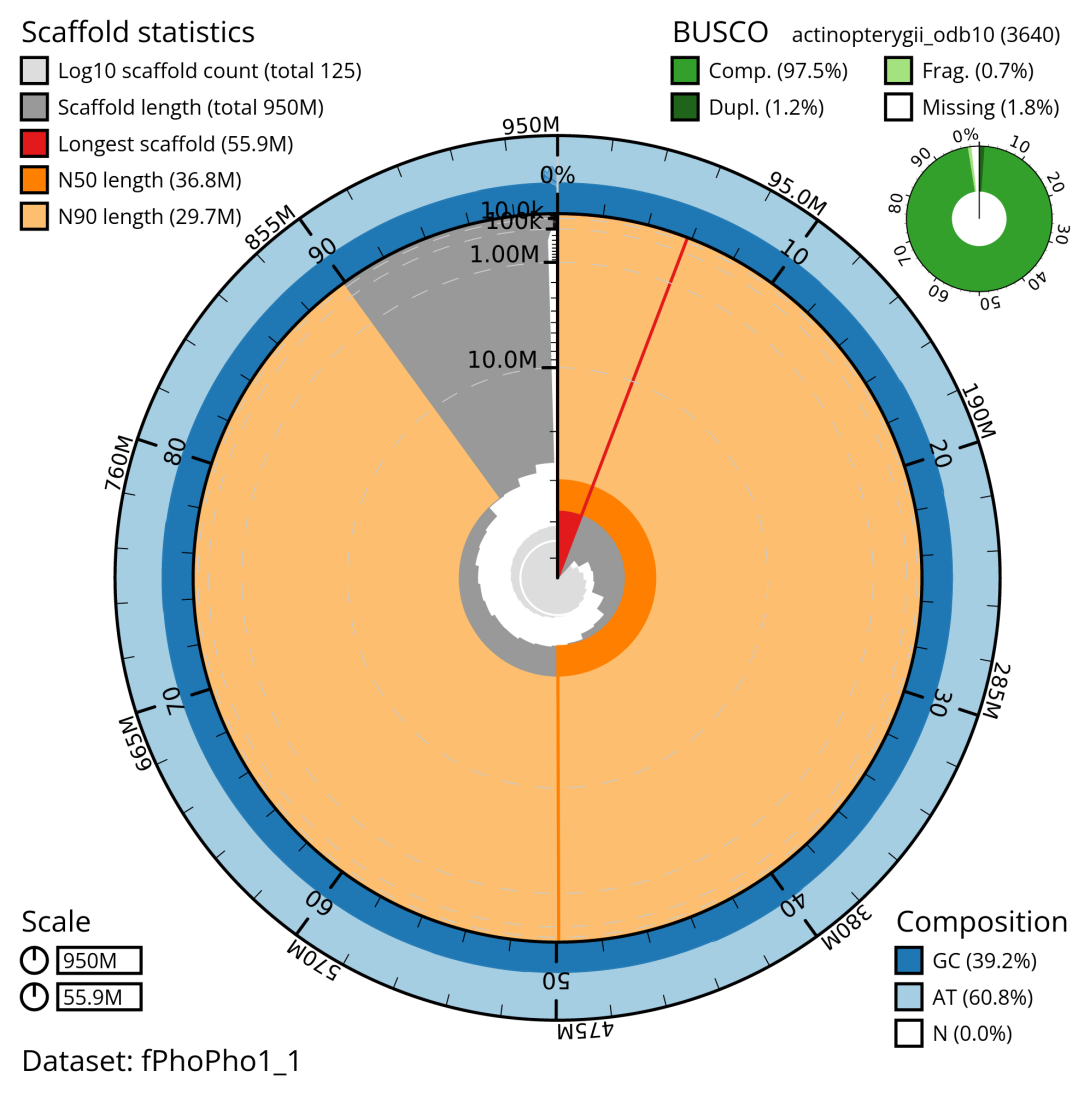
Genome assembly of *Phoxinus phoxinus*, fPhoPho1.1: metrics. The BlobToolKit snail plot shows N50 metrics and BUSCO gene completeness. The main plot is divided into 1,000 size-ordered bins around the circumference with each bin representing 0.1% of the 950,498,056 bp assembly. The distribution of scaffold lengths is shown in dark grey with the plot radius scaled to the longest scaffold present in the assembly (55,893,807 bp, shown in red). Orange and pale-orange arcs show the N50 and N90 scaffold lengths (36,753,459 and 29,721,935 bp), respectively. The pale grey spiral shows the cumulative scaffold count on a log scale with white scale lines showing successive orders of magnitude. The blue and pale-blue area around the outside of the plot shows the distribution of GC, AT and N percentages in the same bins as the inner plot. A summary of complete, fragmented, duplicated and missing BUSCO genes in the actinopterygii_odb10 set is shown in the top right. An interactive version of this figure is available at https://blobtoolkit.genomehubs.org/view/fPhoPho1_1/dataset/fPhoPho1_1/snail.

**Figure 3.**
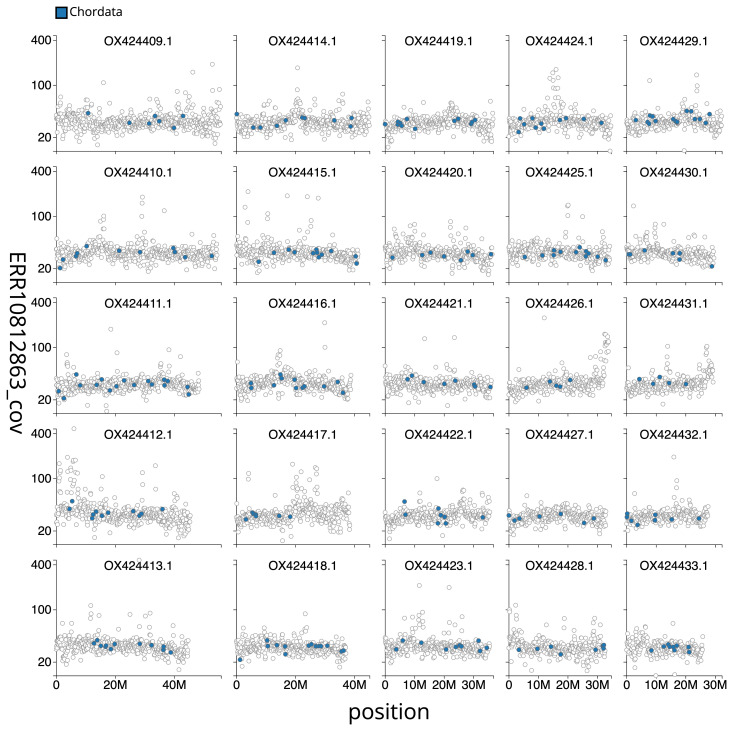
Genome assembly of *Phoxinus phoxinus*, fPhoPho1.1: Distribution plot of base coverage in ERR10812863 against position for sequences in assembly fPhoPho1.1. Windows of 100 kb are coloured by phylum. The assembly has been filtered to exclude sequences with length < 2,550,000. An interactive version of this figure is available here.

**Figure 4.**
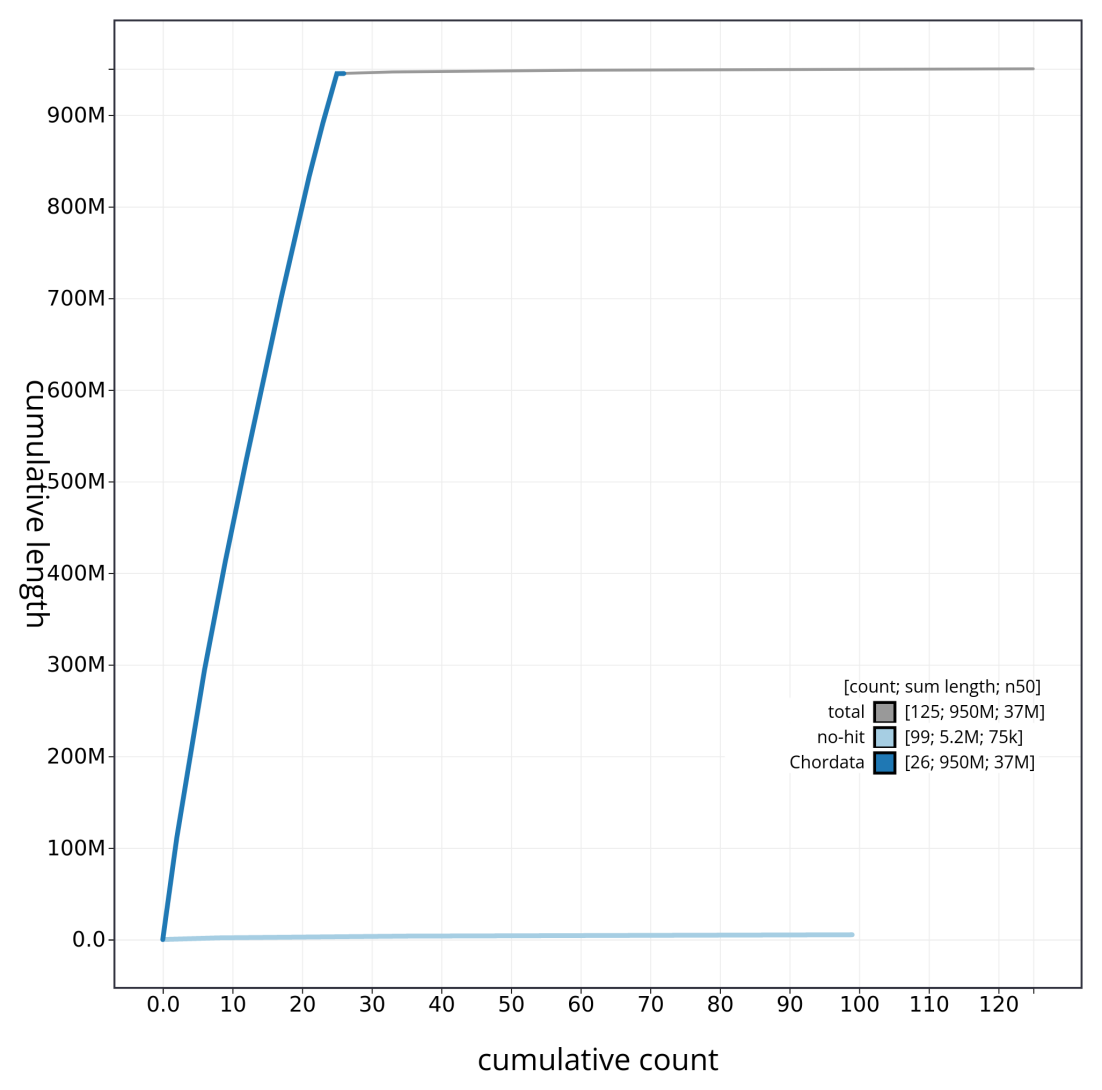
Genome assembly of *Phoxinus phoxinus* fPhoPho1.1: BlobToolKit cumulative sequence plot. The grey line shows cumulative length for all sequences. Coloured lines show cumulative lengths of sequences assigned to each phylum using the buscogenes taxrule. An interactive version of this figure is available at https://blobtoolkit.genomehubs.org/view/fPhoPho1_1/dataset/fPhoPho1_1/cumulative.

**Figure 5.**
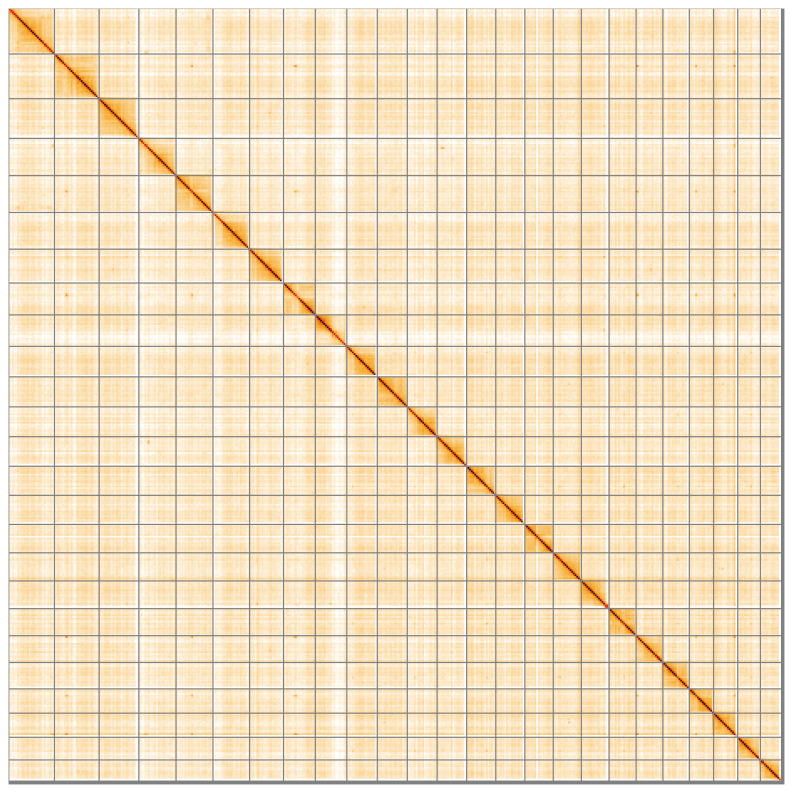
Genome assembly of *Phoxinus phoxinus* fPhoPho1.1: Hi-C contact map of the fPhoPho1.1 assembly, visualised using HiGlass. Chromosomes are shown in order of size from left to right and top to bottom. An interactive version of this figure may be viewed at https://genome-note-higlass.tol.sanger.ac.uk/l/?d=JplTDH_LQAqhxfL1doUPtg.

Manual assembly curation corrected 103 missing joins or mis-joins and 47 haplotypic duplications, reducing the assembly length by 2.5% and the scaffold number by 24.7%, and decreasing the scaffold N50 by 1.4%. The final assembly has a total length of 950.50 Mb in 124 sequence scaffolds with a scaffold N50 of 36.8 Mb ( Table 2). The snail plot in Figure 2 provides a summary of the assembly statistics, while the distribution of base coverage against position per chromosome is shown in Figure 3. The cumulative assembly plot in Figure 4 shows curves for subsets of scaffolds assigned to different phyla. Most (99.48%) of the assembly sequence was assigned to 25 chromosomal-level scaffolds. Chromosome-scale scaffolds confirmed by the Hi-C data are named in order of size ( Figure 5; Table 3). While not fully phased, the assembly deposited is of one haplotype. Contigs corresponding to the second haplotype have also been deposited. The mitochondrial genome was also assembled and can be found as a contig within the multifasta file of the genome submission.

**Table 2.** Genome assembly data for *Phoxinus phoxinus*, fPhoPho1.1.

Genome assembly		
Assembly name	fPhoPho1.1	
Assembly accession	GCA_949152265.1	
*Accession of alternate haplotype*	*GCA_949152275.1*	
Span (Mb)	950.50	
Number of contigs	1,006	
Contig N50 length (Mb)	2.0	
Number of scaffolds	124	
Scaffold N50 length (Mb)	36.8	
Longest scaffold (Mb)	55.89	
Assembly metrics *		*Benchmark*
Consensus quality (QV)	57.1	*≥ 50*
*k*-mer completeness	99.99%	*≥ 95%*
BUSCO **	C:97.5%[S:96.3%,D:1.2%], F:0.7%,M:1.8%,n:3,640	*C ≥ 95%*
Percentage of assembly mapped to chromosomes	99.48%	*≥ 95%*
Sex chromosomes	Not identified	*localised * *homologous pairs*
Organelles	Mitochondrial genome: 18.36 kb	*complete single * *alleles*

* Assembly metric benchmarks are adapted from column VGP-2020 of “Table 1: Proposed standards and metrics for defining genome assembly quality” from Rhie *et al.* (2021).

** BUSCO scores based on the actinopterygii_odb10 BUSCO set using version 5.3.2. C = complete [S = single copy, D = duplicated], F = fragmented, M = missing, n = number of orthologues in comparison. A full set of BUSCO scores is available at https://blobtoolkit.genomehubs.org/view/fPhoPho1_1/dataset/fPhoPho1_1/busco.

**Table 3.** Chromosomal pseudomolecules in the genome assembly of *Phoxinus phoxinus*, fPhoPho1.

INSDC accession	Name	Length (Mb)	GC%
OX424409.1	1	55.89	39.5
OX424410.1	2	54.67	39.5
OX424411.1	3	48.54	38.5
OX424412.1	4	45.53	40.0
OX424413.1	5	44.82	39.0
OX424414.1	6	45.16	39.0
OX424415.1	7	41.35	39.0
OX424416.1	8	38.98	39.5
OX424417.1	9	38.55	40.0
OX424418.1	10	37.2	39.0
OX424419.1	11	36.75	39.0
OX424420.1	12	36.46	39.5
OX424421.1	13	36.15	39.0
OX424422.1	14	35.61	39.0
OX424423.1	15	35.53	39.0
OX424424.1	16	34.73	39.0
OX424425.1	17	34.38	39.0
OX424426.1	18	33.91	39.5
OX424427.1	19	33.06	39.0
OX424428.1	20	32.77	39.0
OX424429.1	21	32.32	39.0
OX424430.1	22	29.72	39.0
OX424431.1	23	29.46	39.0
OX424432.1	24	27.72	39.0
OX424433.1	25	25.94	39.5
OX424434.1	MT	0.02	42.5

The estimated Quality Value (QV) of the final assembly is 57.1 with *k*-mer completeness of 99.99%, and the assembly has a BUSCO v5.3.2 completeness of 97.5% (single = 96.3%, duplicated = 1.2%), using the actinopterygii_odb10 reference set ( *n* = 3,640).

Metadata for specimens, BOLD barcode results, spectra estimates, sequencing runs, contaminants and pre-curation assembly statistics are given at https://tolqc.cog.sanger.ac.uk/darwin/fish/Phoxinus_phoxinus/.

## Methods

### Sample acquisition and nucleic acid extraction

A female specimen of *P. phoxinus* (specimen ID SAN0000705, individual ToLID fPhoPho1) was collected from the River Wharfe, UK (latitude 53.91, longitude –1.61) on 2020-09-09. The specimen was collected by electrofishing by Andy Nunn and Paolo Moccetti, who also formally identified the species. The specimen was transported alive to the University of Hull and left to recover fully in an aquarium before any sampling commenced. The specimen was euthanised in a lethal dose of MS-222. Tissue dissection was carried out by Bernd Hänfling within 30 minutes of euthanasia, and the sampled tissues were immediately shock-frozen in liquid nitrogen.

The workflow for high molecular weight (HMW) DNA extraction at the Wellcome Sanger Institute (WSI) Tree of Life Core Laboratory includes a sequence of core procedures: sample preparation; sample homogenisation, DNA extraction, fragmentation, and clean-up. In sample preparation, the fPhoPho1 sample was weighed and dissected on dry ice ( Jay *et al.*, 2023). Tissue from the spleen was homogenised using a PowerMasher II tissue disruptor ( Denton *et al.*, 2023a).

HMW DNA was extracted using the Automated MagAttract v1 protocol ( Sheerin *et al.*, 2023). DNA was sheared into an average fragment size of 12–20 kb in a Megaruptor 3 system with speed setting 30 ( Todorovic *et al.*, 2023). Sheared DNA was purified by solid-phase reversible immobilisation ( Strickland *et al.*, 2023): in brief, the method employs AMPure PB beads to eliminate shorter fragments and concentrate the DNA. The concentration of the sheared and purified DNA was assessed using a Nanodrop spectrophotometer and Qubit Fluorometer using the Qubit dsDNA High Sensitivity Assay kit. Fragment size distribution was evaluated by running the sample on the FemtoPulse system.

RNA was extracted from muscle tissue of fPhoPho1 in the Tree of Life Laboratory at the WSI using the RNA Extraction: Automated MagMax™ *mir*Vana protocol ( do Amaral *et al.*, 2023). The RNA concentration was assessed using a Nanodrop spectrophotometer and a Qubit Fluorometer using the Qubit RNA Broad-Range Assay kit. Analysis of the integrity of the RNA was done using the Agilent RNA 6000 Pico Kit and Eukaryotic Total RNA assay.

Protocols developed by the WSI Tree of Life laboratory are publicly available on protocols.io ( Denton *et al.*, 2023b).

### Sequencing

Pacific Biosciences HiFi circular consensus DNA sequencing libraries were constructed according to the manufacturers’ instructions. Poly(A) RNA-Seq libraries were constructed using the NEB Ultra II RNA Library Prep kit. DNA and RNA sequencing was performed by the Scientific Operations core at the WSI on Pacific Biosciences Sequel IIe (HiFi) and Illumina NovaSeq 6000 (RNA-Seq) instruments. Hi-C data were also generated from gill tissue of fPhoPho1 using the Arima-HiC v2 kit. The Hi-C sequencing was performed using paired-end sequencing with a read length of 150 bp on the Illumina NovaSeq 6000 instrument.

### Genome assembly, curation and evaluation

***Assembly***

The original assembly of HiFi reads was performed using Hifiasm ( Cheng *et al.*, 2021) with the --primary option. Haplotypic duplications were identified and removed with purge_dups ( Guan *et al.*, 2020). Hi-C reads were further mapped with bwa-mem2 ( Vasimuddin *et al.*, 2019) to the primary contigs, which were further scaffolded using the provided Hi-C data ( Rao *et al.*, 2014) in YaHS ( Zhou *et al.*, 2023) using the --break option. Scaffolded assemblies were evaluated using Gfastats ( Formenti *et al.*, 2022), BUSCO ( Manni *et al.*, 2021) and MERQURY.FK ( Rhie *et al.*, 2020).

The mitochondrial genome was assembled using MitoHiFi ( Uliano-Silva *et al.*, 2023), which runs MitoFinder ( Allio *et al.*, 2020) and uses these annotations to select the final mitochondrial contig and to ensure the general quality of the sequence.

***Assembly curation***

The assembly was decontaminated using the Assembly Screen for Cobionts and Contaminants (ASCC) pipeline (article in preparation). Flat files and maps used in curation were generated in TreeVal ( Pointon *et al.*, 2023). Manual curation was primarily conducted using PretextView ( Harry, 2022), with additional insights provided by JBrowse2 ( Diesh *et al.*, 2023) and HiGlass ( Kerpedjiev *et al.*, 2018). Scaffolds were visually inspected and corrected as described by Howe *et al*. (2021). Any identified contamination, missed joins, and mis-joins were corrected, and duplicate sequences were tagged and removed. The entire process is documented at https://gitlab.com/wtsi-grit/rapid-curation (article in preparation).

***Evaluation of the final assembly***

A Hi-C map for the final assembly was produced using bwa-mem2 ( Vasimuddin *et al.*, 2019) in the Cooler file format ( Abdennur & Mirny, 2020). To assess the assembly metrics, the *k*-mer completeness and QV consensus quality values were calculated in Merqury ( Rhie *et al.*, 2020). This work was done using the “sanger-tol/readmapping” ( Surana *et al.*, 2023a) and “sanger-tol/genomenote” ( Surana *et al.*, 2023b) pipelines. The genome readmapping pipelines were developed using the nf-core tooling ( Ewels *et al.*, 2020), use MultiQC ( Ewels *et al.*, 2016), and make extensive use of the Conda package manager, the Bioconda initiative ( Grüning *et al.*, 2018), the Biocontainers infrastructure ( da Veiga Leprevost *et al.*, 2017), and the Docker ( Merkel, 2014) and Singularity ( Kurtzer *et al.*, 2017) containerisation solutions. The genome was also analysed within the BlobToolKit environment ( Challis *et al.*, 2020) and BUSCO scores ( Manni *et al*., 2021; Simão *et al*., 2015) were calculated.

Table 4 contains a list of relevant software tool versions and sources.

**Table 4.** Software tools: versions and sources.

Software tool	Version	Source
BlobToolKit	4.2.1	https://github.com/blobtoolkit/blobtoolkit
BUSCO	5.3.2	https://gitlab.com/ezlab/busco
bwa-mem2	2.2.1	https://github.com/bwa-mem2/bwa-mem2
Gfastats	1.3.6	https://github.com/vgl-hub/gfastats
Hifiasm	0.16.1-r375	https://github.com/chhylp123/hifiasm
HiGlass	1.11.6	https://github.com/higlass/higlass
Merqury.FK	d00d98157618f4e8d1a91 90026b19b471055b22e	https://github.com/thegenemyers/MERQURY.FK
MitoHiFi	2	https://github.com/marcelauliano/MitoHiFi
PretextView	0.2	https://github.com/wtsi-hpag/PretextView
purge_dups	1.2.3	https://github.com/dfguan/purge_dups
sanger-tol/genomenote	v1.0	https://github.com/sanger-tol/genomenote
sanger-tol/readmapping	1.1.0	https://github.com/sanger-tol/readmapping/tree/1.1.0
YaHS	1.2a	https://github.com/c-zhou/yahs

### Wellcome Sanger Institute – Legal and Governance

The materials that have contributed to this genome note have been supplied by a Darwin Tree of Life Partner. The submission of materials by a Darwin Tree of Life Partner is subject to the **‘Darwin Tree of Life Project Sampling Code of Practice’**, which can be found in full on the Darwin Tree of Life website here. By agreeing with and signing up to the Sampling Code of Practice, the Darwin Tree of Life Partner agrees they will meet the legal and ethical requirements and standards set out within this document in respect of all samples acquired for, and supplied to, the Darwin Tree of Life Project.

Further, the Wellcome Sanger Institute employs a process whereby due diligence is carried out proportionate to the nature of the materials themselves, and the circumstances under which they have been/are to be collected and provided for use. The purpose of this is to address and mitigate any potential legal and/or ethical implications of receipt and use of the materials as part of the research project, and to ensure that in doing so we align with best practice wherever possible. The overarching areas of consideration are:

•     Ethical review of provenance and sourcing of the material

•     Legality of collection, transfer and use (national and international)

Each transfer of samples is further undertaken according to a Research Collaboration Agreement or Material Transfer Agreement entered into by the Darwin Tree of Life Partner, Genome Research Limited (operating as the Wellcome Sanger Institute), and in some circumstances other Darwin Tree of Life collaborators.

## Data Availability

European Nucleotide Archive: *Phoxinus phoxinus* (Eurasian minnow). Accession number PRJEB59308; https://identifiers.org/ena.embl/PRJEB59308 ( Wellcome Sanger Institute, 2023). The genome sequence is released openly for reuse. The *Phoxinus phoxinus* genome sequencing initiative is part of the Darwin Tree of Life (DToL) project. All raw sequence data and the assembly have been deposited in INSDC databases. The genome will be annotated using available RNA-Seq data and presented through the Ensembl pipeline at the European Bioinformatics Institute. Raw data and assembly accession identifiers are reported in Table 1 and Table 2.

## Author information

Members of the Wellcome Sanger Institute Tree of Life Management, Samples and Laboratory team are listed here: https://doi.org/10.5281/zenodo.12162482.

Members of Wellcome Sanger Institute Scientific Operations: Sequencing Operations are listed here: https://doi.org/10.5281/zenodo.12165051.

Members of the Wellcome Sanger Institute Tree of Life Core Informatics team are listed here: https://doi.org/10.5281/zenodo.12160324.

Members of the Tree of Life Core Informatics collective are listed here: https://doi.org/10.5281/zenodo.12205391.

Members of the Darwin Tree of Life Consortium are listed here: https://doi.org/10.5281/zenodo.4783558.
