# Supplementary material for: The genome sequence of the Eurasian minnow, Phoxinus phoxinus (Linnaeus, 1758)
Source: Wellcome Open Res. 2024 Sep 3;9:504. [Version 1] doi: 10.12688/wellcomeopenres.22867.1 (PMC11599798; doi:10.12688/wellcomeopenres.22867.1)
Supplement: Peer review history

# Reviewer report: Reviewer response for version 1

**DOI:** 10.21956/wellcomeopenres.25180.r111157

**Published:** 2024-11-26

**Authors:** Matthias Stöck

**Affiliations:** 1 Leibniz-Institute of Freshwater Ecology and Inland Fisheries - IGB, Berlin, Germany

In general, this is a sufficient genome paper to provide a chromosome-scale genome of one taxon among the European minnows. Although a reference genome is usually based on a single specimen from a certain locality, and while the authors acknowledge the “complex and unresolved” taxonomy, they should in more detail refer to the phylogeographic diversity within Europe, namely, e.g. the paper by Palandačić et al. (2020, https://doi.org/10.1038/s41437-019-0292-1).

 Likewise, the current Wellcome Open Research paper does not cite the (peer reviewed by PCI Genomics) pre-publication: https://www.biorxiv.org/content/10.1101/2023.11.30.569369v7 that likewise published a chromosome-scale *Phoxinus phoxinus* genome that should be discussed.

 Technically, the contig N50 of only 2 Mbp are smaller than expected for HiFi-reads but should nevertheless be reported and it should be explained in the text, what has limited the contig size.

 The following two sentences contain from my perspective an error since only full phasing would allow assembling a single chromosome-wide haplotype:

 “While not fully phased, the assembly deposited is of one haplotype. Contigs corresponding to the second haplotype have also been deposited.”

 Furthermore, the photo-voucher looks technically not convincing, ideally fins should be stretched etc., the two metric scales under the specimen look somewhat arbitrary etc. but I fear this cannot be corrected at this point.

Are sufficient details of methods and materials provided to allow replication by others?

Yes

Is the rationale for creating the dataset(s) clearly described?

Yes

Are the datasets clearly presented in a useable and accessible format?

Yes

Are the protocols appropriate and is the work technically sound?

Yes

Reviewer Expertise:

Evolutionary biology, genomics, sex determination of vertebrates

I confirm that I have read this submission and believe that I have an appropriate level of expertise to confirm that it is of an acceptable scientific standard.

## References

[1] : European minnows through time: museum collections aid genetic assessment of species introductions in freshwater fishes (Cyprinidae: Phoxinus species complex). *Heredity (Edinb)* .2020;124(3) : 10.1038/s41437-019-0292-1 410-422 10.1038/s41437-019-0292-1 31896822 PMC7028953

[2] : A chromosome-level, haplotype-resolved genome assembly and annotation for the Eurasian minnow (Leuciscidae: Phoxinus phoxinus) provide evidence of haplotype diversity. *bioRxiv* .2023; 10.1101/2023.11.30.569369 10.1101/2023.11.30.569369 PMC1177547039877992

# Reviewer report: Reviewer response for version 1

**DOI:** 10.21956/wellcomeopenres.25180.r108712

**Published:** 2024-11-21

**Authors:** Radka Symonova

**Affiliations:** 1 Department of Computer Science, Faculty of Science, University of South Bohemia, Institute of Hydrobiology, Biology Centre, Czech Academy of Sciences, Na Sádkách, Czech Republic

I am really happy I do not need to repeat the manuscript description and comments raised by the other reviewer, this is very helpful and many thanks for it!

 The only one point that came to my mind regarding this manuscript is that the authors did not utilize any cytological data known for the species under study. They could have done this easily at the animal genome size database genomesize.com and they could have embedded their results into this context which is quite desirable.

Are sufficient details of methods and materials provided to allow replication by others?

Yes

Is the rationale for creating the dataset(s) clearly described?

Yes

Are the datasets clearly presented in a useable and accessible format?

Yes

Are the protocols appropriate and is the work technically sound?

Yes

Reviewer Expertise:

animal genome evolution focused on the AT/GC compositional aspects in fishes

I confirm that I have read this submission and believe that I have an appropriate level of expertise to confirm that it is of an acceptable scientific standard.

# Reviewer report: Reviewer response for version 1

**DOI:** 10.21956/wellcomeopenres.25180.r99707

**Published:** 2024-10-08

**Authors:** Steven M Mussmann

**Affiliations:** 1 Abernathy Fish Technology Center, U.S. Fish and Wildlife Service, Longview, Washington, USA

This manuscript describes the genome assembly of an individual *Phoxinus phoxinus* (Eurasian minnow). The authors provide basic life history traits, habitat preferences, and morphological characteristics of the species. It is listed as “least concern” by IUCN but still faces many environmental pressures. The authors assembled a nuclear genome from a single female specimen, which adds to two existing genome assemblies for this species. PacBio and Hi-C data were utilized. Laboratory and bioinformatic procedures seem to follow established protocols. RNA data were produced from a single tissue type (gill) and few reads were obtained (~77.4 million reads, 11.69 Gb). Metrics used to report contiguity and completeness of the genome are fairly standard, but could benefit from a little more detail (see “Required items to address”). Overall, the data note is well written and the authors seem to have assembled a high quality genome. However, I have a few relatively minor comments for them to address. These are the reasons I answered ‘partly’ to the questions concerning whether sufficient details of methods and materials provided, and whether the work was technically sound:

**Required items to address:**

- The assembly is based on one female specimen from the River Wharfe, UK, and the authors provide latitude and longitude for the collection site. The authors note that “The current species name probably includes more than one distinct taxonomic entity.” Is the locality description (River Wharfe) adequate for other researchers to determine the “distinct taxonomic entity” to which this individual may belong?

 

- How many SMRT cells were used for sequencing?

 

- Table 2 – it would also be nice to include N90, L50, and L90. It seems from the rest of the data provided that these statistics will also likely indicate high contiguity of the genome, but having them available would facilitate comparisons with other researchers’ work. Some of this information appears in Figure 2 but may be more accessible in Table 2.

 

- The RNA data seem inadequate for annotation of the genome (~77.4 million reads from a single tissue type). Perhaps the authors could address the intended purpose of these data. Alternatively, the authors barely mention these data in their data note so perhaps they could be removed entirely.

**Optional:**

- I have seen many genome reports assess repeat content. After looking at some other data notes for Wellcome Open Research I see this is not a standard item for inclusion in their reports, but it could be interesting to provide which would facilitate comparisons to other species’ genomes.

 

- I was surprised at the length of the mtDNA genome for this species and discovered when searching genbank that mitogenomes for this species seem to vary in lengthy by about 2kbp. It could be interesting to compare these genomes to identify the region(s) responsible for this size discrepancy.

Are sufficient details of methods and materials provided to allow replication by others?

Partly

Is the rationale for creating the dataset(s) clearly described?

Yes

Are the datasets clearly presented in a useable and accessible format?

Yes

Are the protocols appropriate and is the work technically sound?

Partly

Reviewer Expertise:

Population genomics, phylogenetics, molecular ecology.

I confirm that I have read this submission and believe that I have an appropriate level of expertise to confirm that it is of an acceptable scientific standard, however I have significant reservations, as outlined above.
